# Genetic diversity of arsenic accumulation in rice and QTL analysis of methylated arsenic in rice grains

**DOI:** 10.1186/1939-8433-6-3

**Published:** 2013-01-11

**Authors:** Masato Kuramata, Tadashi Abe, Akira Kawasaki, Kaworu Ebana, Taeko Shibaya, Masahiro Yano, Satoru Ishikawa

**Affiliations:** 1 Soil Environment Division, National Institute for Agro-Environmental Sciences, 3-1-3 Kannondai, Tsukuba, Ibaraki, 305-8604 Japan; 2 Genetic Resources Center, National Institute of Agrobiological Sciences, 2-1-2 Kannondai, Tsukuba, Ibaraki, 305-8602 Japan; 3 Agrogenomics Research Center, National Institute of Agrobiological Sciences, 2-1-2 Kannondai, Tsukuba, Ibaraki, 305-8602 Japan

**Keywords:** Dimethylarsinic acid, Inorganic arsenic, QTL, World rice core collection

## Abstract

**Background:**

Rice is a major source of dietary intake of arsenic (As) for the populations that consume rice as a staple food. Therefore, it is necessary to reduce the As concentration in rice to avoid the potential risk to human health. In this study, the genetic diversity in As accumulation and As speciation in rice grains was investigated using a world rice core collection (WRC) comprising 69 accessions grown over a 3-year period. Moreover, quantitative trait locus (QTL) analysis was conducted to identify QTLs controlling the dimethylarsinic acid (DMA) content of rice grains.

**Results:**

There was a 3-fold difference in the grain As concentration of WRC. Concentrations of total-As, inorganic As, and DMA were significantly affected by genotype, year, and genotype-year interaction effects. Among the WRC accessions, Local Basmati and Tima (*indica* type) were identified as cultivars with the lowest stable total-As and inorganic As concentrations. Using an F_2_ population derived from Padi Perak (a high-DMA accession) and Koshihikari (a low-DMA cultivar), we identified two QTLs on chromosome 6 (*qDMAs6.1* and *qDMAs6.2*) and one QTL on chromosome 8 (*qDMAs8*) that were responsible for variations in the grain DMA concentration. Approximately 73% of total phenotypic variance in DMA was explained by the three QTLs.

**Conclusions:**

Based on the results provided, one strategy for developing rice cultivars with a low level of toxic As would be to change the proportion of organic As on the basis of a low level of total As content.

**Electronic supplementary material:**

The online version of this article (doi:10.1186/1939-8433-6-3) contains supplementary material, which is available to authorized users.

## Background

Arsenic (As) exists in the natural environment and food in various chemical forms, which are mainly classified into two groups: organic As and inorganic As (iAs). Although iAs is more toxic than the organic, methylated species (Cullen and Reimer 1989), recent studies indicate that trivalent methylated arsenicals have higher cytotoxicity than pentavalent methylated arsenicals and iAs (Styblo et al.^2000^; Markley and Herbert 2009). The concentrations and proportions of organic As and iAs varies depending on the food type (Signes-Pastor et al.^2008^; EFSA^2009^).

Rice is a major contributor to As intake in humans (Williams et al.^2005^; Meharg et al.^2009^; Gilbert-Diamond et al.^2011^), because rice is mainly cultivated in anaerobic paddy soil, where arsenite [As(III)] is more available (Takahashi et al.^2004^). The four main As species are found in rice grains are As(III), arsenate [As(V)], monomethylarsonic acid (MMA), and dimethylarsinic acid (DMA) (Williams et al.^2005^). Arsenic species play a major role in determining the amount of As absorbed after consumption of As-contaminated rice. DMA in rice is poorly absorbed *in vivo* after oral administration, resulting in a low bioavailability in the human body. Conversely, iAs in rice are much more bioavailable than DMA, indicating a high potential risk to human health (Juhasz et al.^2006^). The factors controlling the total As concentration and the proportions of As species in the rice grain are complicated; genetics is a factor, because there is large variability in phenotypes among different rice cultivars (Norton et al.2009b; Pillai et al.^2010^; Norton et al.^2012^). However, the genetic diversity in total-As and As species of rice germplasms is not fully understood, because previous studies used relatively small numbers of cultivar sets and cultivars with similar genetic backgrounds. A world rice core collection (WRC) comprising 69 accessions, which covers the genetic diversity of almost 32,000 accessions of Asian cultivated rice (*Oryza sativa* L.), has been developed to explore the genetic diversity of various traits (Kojima et al.^2005^). Using the WRC, we found a large genotypic variation in grain Cd concentration (Uraguchi et al.^2009^) and succeeded in identifying the quantitative trait loci (QTLs) controlling Cd accumulation in rice (Abe et al.^2011^). Therefore, the WRC is a powerful tool for evaluating the genetic diversity in total As and As speciation in rice grains, and for further genetic analysis.

The selection of rice cultivars with low As is an effective approach for reducing the As contamination in rice. Moreover, information on the genetic control of As concentration in rice is required for the efficient breeding of commercial cultivars possessing both low-As and good agronomic traits. A previous study showed that QTLs related to total As in rice grains were found on chromosome 6 and 8 in a population derived from low- (cv. CJ06) and high-As accumulating cultivars (cv. TN1) (Zhang et al.^2008^). Although both As(III) and methylated As (MMA and DMA) are taken up by the rice roots through the Lsi1 transporter of silicic acid (Si) (Ma et al.^2008^; Li et al.^2009^), the dynamics of each As species in the plant body appears to be different (Carey et al.^2010^; Kuramata et al.^2011^). As far as we know, QTLs related to each As species in rice grains, especially DMA concentration, have not yet been studied.

The objectives of this study were 1) to evaluate the genetic diversity in total As and As speciation in rice grains of the WRC, and 2) to identify QTLs associated with the DMA concentration in rice. The exploitation of rice cultivars with a higher proportion of DMA than iAs would reduce the risk to human health.

## Results

### Variation in grain As concentrations in WRC

The WRC set, comprising 69 accessions, was cultivated in a paddy field with naturally abundant level of As. Some rice cultivars did not attain full maturity because of a delay in heading at the experimental site; therefore, only 58 rice cultivars were used in this study and were cultivated repeatedly across 3 years.

Two-way ANOVA indicated that total-As, iAs (sum of arsenite and arsenate), and DMA concentrations were significantly affected by genotype (G), year (Y), and G × Y factors (Table 1). Total-As largely differed by G and Y factors, explaining 35.1 and 47.8% of the total variation, respectively. A similar trend was observed for iAs. A larger contribution of G (62.3%) than Y (7.2%) and G × Y (27.9%) factors was found for the DMA concentration, indicating that the differences in grain DMA mostly depend on genotype.

**Table 1:** **Two-way ANOVA of As species in brown rice of WRC cultivars grown over a 3-year period in a paddy field under flooded conditions**

As species	Variation	SS	***df***	MS	***F***	***P***	SSE/SST^a^ (%)
Total As	G	0.4980	57	0.0087	56.72	<0.0001	35.1
	Y	0.6771	2	0.3386	2197.9	<0.0001	47.8
	G x Y	0.2160	114	0.0019	12.3	<0.0001	15.2
	Error	0.0268	174	0.0002			
iAs	G	0.3641	57	0.0064	139.00	<0.0001	42.0
	Y	0.3113	2	0.1556	3387.4	<0.0001	36.0
	G x Y	0.1824	114	0.0016	34.83	<0.0001	21.1
	Error	0.0080	174	0.0000			
DMA	G	0.0245	57	0.0004	74.93	<0.0001	62.3
	Y	0.0028	2	0.0014	247.7	<0.0001	7.2
	G x Y	0.0110	114	0.0001	16.77	<0.0001	27.9
	Error	0.0010	174	0.0000			

Total As, total arsenic of grains; iAs, sum of As (III) and As (V); mAs, dimethylarsinic acid; G, Genotype; Y, year; G × Y, genotype-by-year interaction.

^a^ Sum of squares (SS) of each effect by total SS.

**Figure 1:**
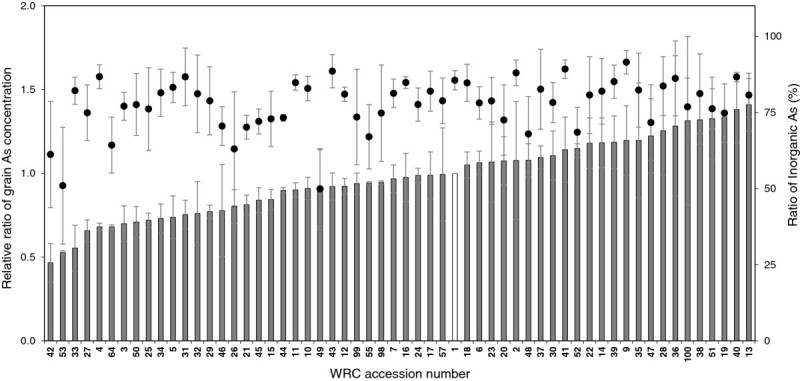
**Genotypic variation of As accumulation in grains of the world rice core collection (WRC) comprising 69 accessions.** The WRC was cultured over 3 years in a paddy field with naturally abundant level of As. The accessions that did not ripen because of late heading were omitted from the As analysis and 58 accessions were presented. The columns indicate the average ratio over 3 years of the total As concentration (filled columns) of each genotype to that of WRC1 (open column), *japonica* cv. Nipponbare. Circles indicate the average proportions of inorganic As (iAs) to sum of As species in each genotype.

The WRC ranking of total As concentrations in grains over 3 years are shown in Figure 1. Total grain As of WRC1 (a reference cultivar), *japonica* type cv. Nipponbare, varied over 3 years, i.e., 0.180 mg kg^-1^ for first year, 0.107 mg kg^-1^ for second year, and 0.206 mg kg^-1^ for third year (Additional file 1). To normalize the differences in total grain As concentrations among years, the ratio of the concentration of each genotype to WRC1 grown in the same year was used for the ranking. WRC42 (cv. Local Basmati) and WRC53 (cv. Tima) were categorized as the lowest total grain As cultivars: the ratio was approximately one-half that of WRC1. On the other hand, WRC13 (cv. Asu) and WRC40 (cv. Nepal 555) showed the highest As concentrations, with a ration 1.4-times higher than that of WRC1. A 3-fold difference in the total grain As concentration was observed between the lowest and highest WRC cultivars.

The genotypic variation of iAs is presented as the proportions of iAs to sums of iAs and DMA in the WRC grains (Figure 1, circles). The proportions of iAs ranged from 49.9% to 91.5% and iAs was the predominant As species in most cultivars. However, several cultivars presented lower ratios of iAs, around 50–60%. WRC49, cv. Padi Perak, was a typical cultivar having a lower iAs ratio, although the total As concentration in the grains were similar to that of WRC1. This indicates that the DMA concentrations in the grains were higher in WRC49 than in WRC1 (Additional file 1). WRC42 and WRC53 showed the lowest total grain As and were also ranked as lower iAs cultivars.

Total As concentrations were positively correlated with iAs concentrations in the WRC grains grown in 2009 (Additional file 2; *p* < 0.001, *r* = 0.909). This trend was similar in 2007 (*p* < 0.001, *r* = 0.823) and 2008 (*p* < 0.001, *r* = 0.615). On the other hand, the correlation coefficients between total As and DMA were smaller than those of total As and iAs, although statistically significant correlations were observed in 2007 (*p* < 0.005, *r* = 0.385) and 2009 (*p* < 0.05, *r* = 0.270), but not in 2008 (*p* = 0.702, *r* = −0.051).

### As accumulation in plants tissue among genotypes

**Figure 2:**
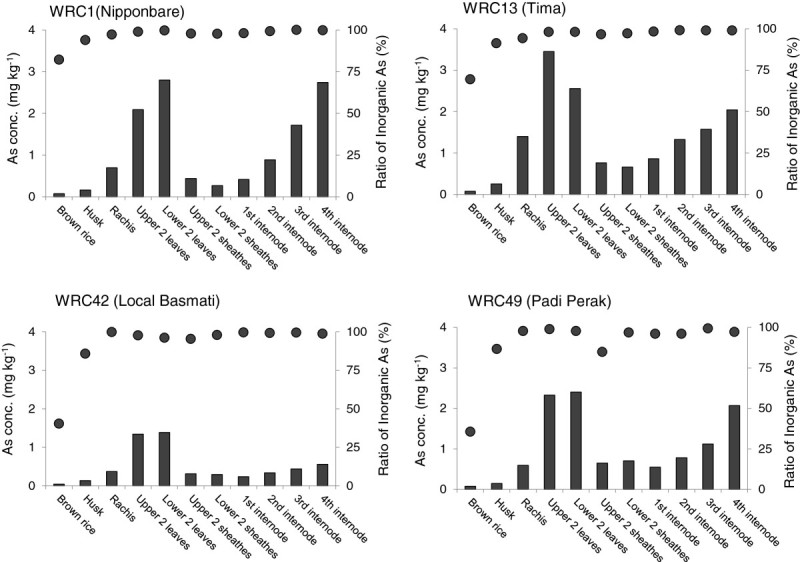
**Total As concentration (filled columns) and the ratio of inorganic As (filled circles) in each plant part of four rice cultivars: a reference cultivar (WRC1, Nipponbare), the lowest total-As cultivar (WRC42, Local Basmati), the highest total-As cultivar (WRC13, Asu), and the highest-DMA cultivar (WRC49, Padi Perak).**

Total concentrations of As and the proportions of each As species in various plant tissues were compared among selected cultivars (WRC1, WRC13, WRC42, and WRC49) that differed in total As or DMA concentrations in their grains (Figure 2). In WRC1, the As concentration of the grain (brown rice) was the lowest, followed by that of the husk. Arsenic accumulated at the highest levels in upper leaf blades and increased with increasing internode number. The As level of the rachis was slightly higher than that of the leaf sheathes. Similar distribution patterns of As accumulation were observed in other selected rice cultivars. All plant parts of WRC42 (a low-As accumulating accession) showed lower As concentrations than the corresponding parts of WRC1. On the other hand, higher As concentrations in the upper leaf blades, rachis, husk, and brown rice were observed in a high-As accumulating cultivar, WRC13. WRC42 and WRC49 had lower proportions of iAs in their grains than WRC1, whereas iAs was present in high proportions in other plant parts.

### QTL analysis for DMA concentrations of rice grains

**Figure 3:**
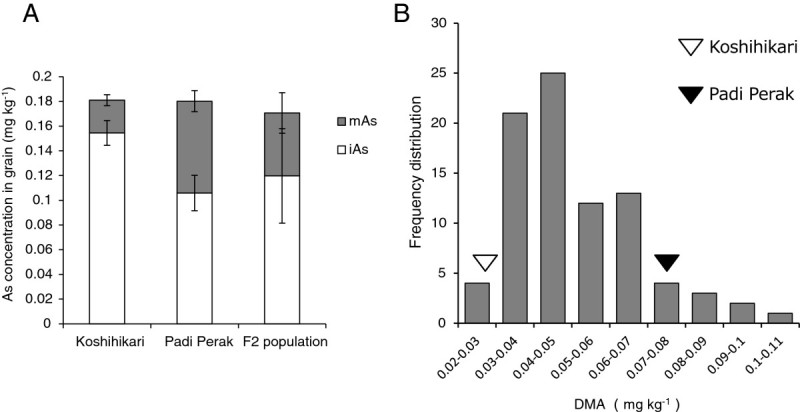
**As concentration in Koshihikari, Padi Perak, and an F**_**2**_**population.** (**A**) Average concentrations of inorganic As (iAs) and DMA in grains. (**B**) Frequency distribution for the grain DMA concentrations in the F_2_ population. Black and white arrows indicate the means of Padi Perak and Koshihikari, respectively.

The QTLs responsible for DMA concentrations in rice grains were investigated. One parental cultivar, Koshihikari, was identical to WRC1 Nipponbare in terms of As concentrations; the iAs concentration was higher than the DMA concentration in the grains (Figure 3A) and the concentrations were 0.155 mg kg^-1^ for iAs and 0.027 mg kg^-1^ for DMA. By contrast, the other parent, WRC49 (Padi Perak), showed 0.106 mg kg^-1^ for iAs and 0.074 mg kg^-1^ for DMA. Thus, Padi Perak had a higher grain DMA concentration than Koshihikari. The average concentrations of iAs and DMA in the F_2_ population were 0.120 mg kg^-1^ and 0.051 mg kg^-1^, respectively, and their values lay between the parental cultivars. A wide and continuous variation in the grain DMA concentrations (0.041–0.351 mg kg^-1^) was observed in the F_2_ population (Figure 3B).

**Figure 4:**
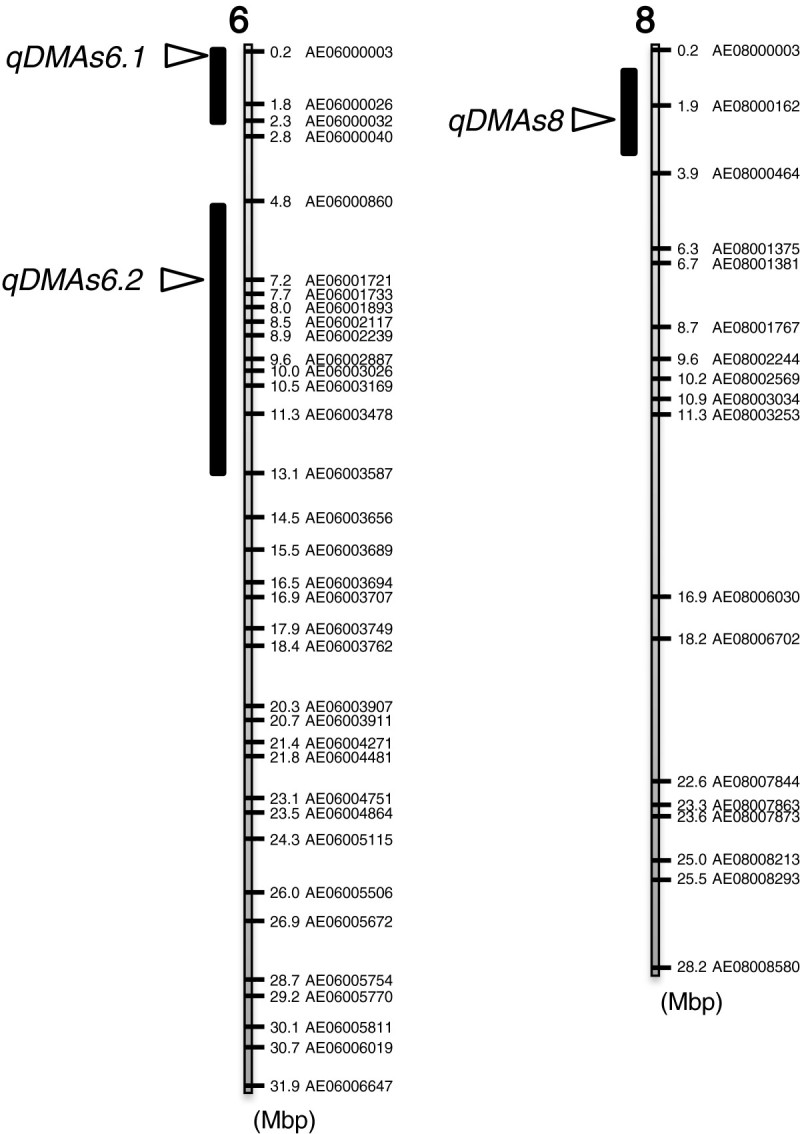
**SNP linkage map showing locations of QTLs related to grain DMA concentration.** Chromosome numbers are indicated above and SNP marker names and physical distance (Mbp) from the distal end of the short arm of each chromosome are shown. The bars and triangle symbols represent chromosome regions exceeding a threshold logarithm of odds (LOD, 3.88) value for the trait, according to a 1000-permutation test and LOD peaks of putative QTLs, respectively.

Three QTLs related to the grain DMA concentrations were identified. A summary of the QTL analysis and the chromosomal locations of the QTLs are shown in Table 2 and Figure 4, respectively. Two QTLs were detected on the short arm of chromosome 6 and were designated tentatively as *qDMAs6.1* and *qDMAs6.2*. The other QTL was located on chromosome 8 and designated tentatively as *qDMAs8*. All QTLs increased the grain DMA concentration, with the additive effects coming from the Padi Perak allele. Approximately 73% of the phenotypic variance was explained by the three QTLs, and *qDMAs6.1*, on chromosome 6 accounted for 36% of the variance.

**Table 2:** **Quantitative trait loci (QTLs) for grain DMA concentration in the F**_**2**_ **population**

Trait	QTL^a^	Chromosome	Nearest maker	LOD^b^	Additive effect	***r*** ^***2*** d^
Grain DMA concentration	*qDMAs6.1*	6	AE06000003	11.06	−0.014	0.36
	*qDMAs6.2*	6	AE06001721	6.90	−0.007	0.25
	*qDMAs8*	8	AE08000162	3.89	−0.0089	0.12

^a^ Individual QTLs are shown in italics as the abbreviation of the trait and the chromosome number.

^b^After 1000-permutation tests, the threshold value of the logarithm of odds (LOD) was calculated as 3.88.

^c^A negative value of the additive effect indicates that the allele from Padi Perak increased the phenotypic value.

^d^*r*^*2*^ represents the proportion of the phenotypic variance explained by the QTL.

## Discussion

In this study, we found that all the factors studied (genotype, year, and genotype by year interaction) affected total-As, iAs, and DMA concentrations in the WRC rice grains (Table 1). Similar findings were reported by (Pillai et al.^2010^), who compared total-As and As species concentration in the grains of approximately 20 rice cultivars grown across 3-years on a flooded paddy field with a moderate As concentration. Relatively large percentages of year-variations compared with the total variations were observed in total-As and iAs concentrations, 47.8 and 36.0%, respectively, even though all the WRC accessions were grown in the same field across 3 years. The concentrations of total-As and iAs in the all WRC grains were significantly lower in 2008 than in 2007 and in 2009 (Additional file 1). The delay of transplanting the plants into the paddy field in 2008 might have affected their As concentrations, especially for iAs, which is the major species in total-As, and resulted in some inconsistency in rankings of cultivars for As concentrations among years. On the other hand, annual differences in DMA concentrations of the WRC grains were small, and the variation was largely attributable to genetics. Therefore, these results indicate that the DMA concentration in the rice grain could be modified through genetic analysis and plant breeding.

A 3-fold difference in the total As of rice grains in the WRC was found (Figure 1). Our previous report showed that there was a 67-fold difference in the grain Cd concentration in the same WRC accessions grown in the same field used in this study (Uraguchi et al.^2009^). The screening results indicated that the genetic diversity of the grain As concentration is not as wide as grain Cd concentrations. In addition, previous reports suggested that the rankings of rice cultivars for total grain As concentrations vary greatly across environments (Norton et al.2009a; Ahmed et al.^2011^), indicating the difficulty of genotype selection for lowering rice grain As concentration. Although we did not confirm the genotypic variation in the WRC grain As concentrations across different sites, two cultivars, WRC42 (Local Basmati) and WRC53 (Tima), showed the lowest concentrations of total-As and iAs in their grains and the concentrations were stably low across the 3 years (Figure 1 and Additional file 1). A cultivar, BR3, adapted for the boro rice season was identified as showing a stable low grain As by two papers, independently (Norton et al.2009b; Ahmed et al.^2011^); the ratios of grain As concentrations of BR3 to that of Nipponbare were 0.58 and 0.66 when their cultivars were grown on two sites in Bangladesh (Norton et al.2009b). In this study, the ratio to Nipponbare was 0.46 for Local Basmati and 0.53 for Tima, suggesting that the two cultivars have an equivalent or superior ability for reducing grain their As concentrations to that of BR3.

It has been suggested that differences in days to heading (DTH) may be associated with the genotypic variation in grain As concentrations (Pillai et al.^2010^). In this study, no correlation between the DTH and total grain As concentrations was found among the WRC accessions (*r* = −0.025, *p* = 0.853 in 2007, *r* = −0.222, *p* = 0.094 in 2008, and *r* = −0.124, *p* = 0.355 in 2009). However, DTH was longer for the low-As cultivars Local Basmati and Tima than for the Nipponbare; differences of approximately 20 days were observed. The ears of low-As cultivars appeared when the temperature gradually decreased at the experimental site, and a relatively lower temperature during heading to maturing could have influenced As transport into their grains. Further investigation is needed to determine the effect of DTH on grain As concentration in rice.

The As concentrations of Local Basmati was the lowest, not only in the grains, but also in other shoot parts (Figure 2). On the other hand, cv. Asu had the highest values for all plant parts. iAs is a major species in all shoot parts; therefore, the differences in total As observed in all plant parts between the cultivars could be explained by the difference in root iAs uptake and/or translocation of iAs from roots to shoots. As(III), the dominant As species in the anaerobic paddy soil, is taken up by rice roots principally through the Lsi1 transporter, which is responsible for Si uptake (Ma et al.^2008^). In addition, the dynamics of As(III) and Si in the plant body after root uptake might be similar, because the highest concentrations and amounts of As (Figure 2) and Si (Ma et al.^1989^) were found in leaf blades in rice. Although iAs was also the dominant species in rice grains of the WRC, the proportions were not in accordance with those observed in other plant parts. DMA accumulated specifically in the grains, and Padi Perak had the highest DMA concentrations and the lowest proportions of iAs in its grains (Figure 1 and Additional file 1). This trend was stable across 3 years, so this cultivar is likely to have a specific ability to accumulate DMA in the grain.

The results of QTL analysis revealed that *qDMAs6.1* and *qDMAs6.2*, on chromosome 6 and *qDMAs8* on chromosome 8 were responsible for increasing the grain DMA concentration and all of these alleles were inherited from Padi Perak (Table 2 and Figure 4). (Zhang et al.^2008^) also identified two QTLs for total grain As concentrations on chromosome 6 and 8. The QTLs were located at 19–24 Mbp on chromosome 6 and at 4.8-5.1 Mbp on chromosome 8 in physical distance (Gramene, http://www.gramene.org/). These QTL positions are different to those identified in the present study. (Norton et al.^2010^) found several QTLs for total As concentrations in rice leaves and a QTL, *qAs6.1*, near marker RM253 was located within our QTL region between AE06000860 and AE06003587. MMA and DMA are also taken up by rice roots through the rice aquaporin Lsi1 (OsNIP2;1) transporter (Li et al.^2009^). However, the differences in grain DMA concentrations between Padi Perak and Koshihikari are not likely to be directly associated with this transporter, because the *Lsi1* gene (Os02g0745100) is located on chromosome 2. Conversely, *qDMAs6.2* contains the *Lsi6* (*OsNIP2;2*) gene, which encodes an Si transporter related to inter-vascular transfer in the nodes of rice plants and is a homolog of the *Lsi1* gene (Yamaji and Ma 2009). Although Lsi6 can also mediate arsenite transport when expressed in *Xenopus* oocytes, knockout lines of *Lsi6* did not show decreased As concentrations in their shoots and roots compared with the wild-type (Ma et al.^2008^). This may be the result of assaying As at the seedling stage; *Lsi6* is specifically expressed in the node I below panicles after the grain filling stage (Yamaji and Ma 2009). If Lsi6 in node I is permeable to methylated As species MMA and DMA, like Lsi1 in the roots, differences in grain DMA concentration in rice cultivars may be caused by a differential transport function of Lsi6 or a differential expression of the *Lsi6* gene. In excised panicles, high mobility of DMA in the xylem and phloem, and specific unloading of DMA into the grain, has been reported by (Carey et al.^2010^).

Several researchers have observed that As methylation may not occur *in planta*, because methylated As species were not found in plants exposed to iAs under axenic conditions (Jia et al.^2012^; Lomax et al.^2012^). In addition, there was no further methylation in rice plants exposed to MMA or DMA under axenic conditions (Jia et al.^2012^). These results suggest that methylated As species present in rice plants are derived from the soil through microorganism-mediated methylation. The microarray-based GeoChip analysis of microbial genes revealed that a methyltransferase gene, *arsM*, was abundant in a Bangladeshi paddy soil containing a high As level (34 mg kg^-1^), suggesting that DMA is produced by specific soil microbes (Lomax et al.^2012^). The abundance of root exudates stimulates microbial growth and attracts more soil microorganisms to the root surface (Marschner et al.^2011^). The density and activity of microbes carrying the *arsM* gene may differ in the root surfaces among rice cultivars. In addition, the genotypic variation in As accumulation and speciation in rice could be influenced by the differential rates of radial oxygen loss from their roots (Wu et al.^2011^). Thus, the events that occur in the rhizosphere are important factors for As accumulation and speciation. It is also possible that the detected QTLs are linked to rhizosphere methylation, which is associated with root biology, such as root exudation or root oxidation. In the future, fine mapping is needed to identify the genes related to grain DMA accumulation and functional analysis will also be required to understand the physiological role of the QTL genes.

## Conclusions

The exploitation of rice cultivars that can accumulate less iAs in the grain is a promising technology for reducing As contamination in rice. We found a unique rice cultivar showing a high proportion of DMA in the grain from the WRC and identified several QTLs controlling rice DMA concentration. This cultivar could be used as a starting material to produce new cultivars with a lower iAs ratio through DNA marker assisted selection.

## Methods

### Plant materials and field experiments

The WRC seeds, comprising 69 cultivars, were obtained from the Genebank of the National Institute of Agrobiological Sciences (URL: http://www.gene.affrc.go.jp/databases-core_collections_wr_en.php). To observe the segregation patterns of grain DMA concentrations in F_2_ populations, F_2_ seeds were obtained by crossing a high-DMA accumulating cultivar WRC 49 (cv. Padi Perak) and a low-DMA accumulating cultivar Koshihikari. The Padi Perak and Koshihikari cultivars are categorized as tropical *japonica* and temperate *japonica* types, respectively. To evaluate the As accumulation and speciation in the grain, the WRC was grown over 3 years (2007, 2008, and 2009) on the same paddy field and the F_2_ population was grown in 2010 following the WRC cultivation. The paddy soil was classified as an alluvial soil; the As concentration was 1.4 mg kg^-1^ dry weight, as determined by 1 M HCl extraction in accordance with the *Agricultural Land Soil Pollution Prevention Law* of Japan.

Surface sterilized seeds of the 69 accessions and the F_2_ population were germinated and grown in nursery boxes filled with artificially prepared fertilized soil (Ponzol No.1; Sumitomo Chemical Co., Ltd., Tokyo, Japan). The field site was an experimental paddy field at the National Institute for Agro-Environmental Sciences (latitude 36 01’ 32.31” N, longitude 104 06’ 23.88” E, altitude 21 m) in Tsukuba, Japan. Seedlings (1-month-old) of the WRC were transplanted into the flooded paddy field with a single plant per hill, spaced at 15 × 30 cm and 12 plants of each cultivar were planted per row, in the middle May in 2007 and 2009 and in early in June in 2008. For the F_2_ population, 100 seedlings were transplanted into the field in the same manner as the WRC, in 2010. A commercial chemical fertilizer was applied at the rate of 50 kg-N ha^-1^, 50kg-P ha^-1^, and 50kg-K ha^-1^ as a base or 20 kg-N ha^-1^ as a side dressing, respectively. Surface irrigation was applied before the mid-season drainage was done for about 1 week in early in July, then a full irrigation condition was applied again until grain harvesting. The central two plants in each row of the WRC were collected for As analysis. All plants of the F_2_ population that reached maturity were harvested.

### Sample preparation for As analysis and speciation

After threshing, rice grains in the WRC and F_2_ individuals were dehusked to obtain brown rice and then ground to a fine powder through 0.5-mm mesh using a stainless steel rotor mill (P-14, Fritsch, Kastl, Germany). For several cultivars showing low- and high-As concentrations in their grains (WRC1, WRC13, WRC42, and WRC49), the mature plants were divided into 11 plant parts: brown rice, husk, rachis, upper leaf blades (including flag leaf), lower leaf blades, upper leaf sheaths, lower leaf sheaths, and the first to fourth internodes of culms. These parts were also ground to a fine powder. Powdered sample (0.5g) was digested in 65 mL polypropylene tubes (DigiTUBEs, GL Science, Tokyo, Japan) with a 5mL of an acid mixture comprising high-grade concentrated HNO_3_ and H_2_O_2_ (5:1, v/v) using the heating block system (Digi-PREP LS, SPS SCIENCE, Canada) at 105°C for 3 hrs. The digested solution was filtered using disposable 0.2-μm PTFE syringe filters after appropriate dilution with Milli-Q water. Two certified standard materials, NIST SRM 1568a rice flour (National Institute of Standards and Technology, Gaithersburg, USA) and NMIJ CRM 7503a rice flour (National Metrology Institute of Japan, Tsukuba, Japan), was used to ensure the precision of the analytical procedure.

To determine As species in grains and shoots, a powdered sample (0.2 g dry weight) was mixed with 1 mL of 0.15 M HNO_3_ in a 15 mL polyethylene tube, and the mixture was heated on an aluminum heating block at 80°C for 2 hrs. The solution obtained was diluted to 5 mL with Milli-Q water and passed through a 0.2-μm PTFE syringe filter before analysis.

### Total As determination and speciation analysis

The concentrations of total As in grains and shoots were determined using inductively coupled plasma mass spectroscopy (ICP-MS, ELAN DRC-e, Perkin-Elmer Sciex, DE). Separation of As(III), As(V), MMA(V), and DMA(V) was performed by HPLC (PU 712i, GL Science, Tokyo, Japan) on a reversed phase ODS-3 column (Kuramata et al.^2011^). The solution was eluted from the column and directly introduced to the ICP-MS and the arsenic signal (m/z 75) was monitored. The rice grains of our samples contained mostly As(III) and DMA(V), with trace amounts of MMA(V) and As(V). In this paper, the sum of As(III) and As(V) is presented as total inorganic As (iAs), because the HNO_3_ used in sample preparation generally acts as an oxidative acid (Arao et al.^2009^). Moreover, MMA was only detected in a few samples at the detection limit (0.02 mg kg^-1^); therefore, only DMA concentration was shown as methylated As.

### Genotyping by single-nucleotide polymorphism (SNP) markers

The DNA was extracted from a piece of leaf blade of the parental cultivars and F_2_ individuals. The 307 SNPs used for the genotyping of the F_2_ population were selected from a 768-plex SNPs set, which was derived from a comparison of whole-genome sequencing between Nipponbare and Khau Mac Kho. SNPs were detected using the GoldenGate BeadArray technology platform and BeadStation 500G system (Illumina, San Diego, CA, USA), according to the manufacturer’s instructions. The SNP markers used in the F_2_ population are shown in Additional files 3 and 4.

### Construction of linkage map and QTL analysis

We constructed the linkage map using MAPMAKER/EXP 3.0 (Lander et al.^1987^). QTL analysis was performed using composite interval mapping, as implemented by the Zmapqtl program (model 6) provided by QTL Cartographer version 2.5 (http://statgen.ncsu.edu/qtlcart/). Genome-wide threshold values (α = 0.05) were used to detect putative QTLs, based on the results of 1000 permutations (Churchill and Doerge 1994).

### Statistical analysis

Expected genotypic variance (G), annually environmental variance (E), and G × E interaction were estimated by two-way ANOVA using the IBM SPSS Statistics package (IBM Japan, Tokyo). The percentage variation explained by each source of variation was calculated for the sum of squares of each effect by total sum of squares, according to the report of (Pillai et al.^2010^).

## Electronic supplementary material

Additional file 1: Inorganic As (iAs), DMA, and total As (tAs) concentrations in the grains of 58 cultivars grown for 3 years. (DOC 102 KB)

Additional file 2: Correlation of total As and each the concentration of each As species in unpolished grains of WRC grown in 2009: filled square, inorganic As (*r* = 0.909, n = 58, *p* < 0.001); open circle, DMA (*r* = 0.270, n = 58, *p* < 0.05). (PPT 137 KB)

Additional file 3: List of 307 SNPs used for genotyping of the F_2_ population derived from a cross between Koshihikari and Padi Perak. (XLS 104 KB)

Additional file 4: Physical map constructed using 307 SNP markers. (PPT 3 MB)

Below are the links to the authors’ original submitted files for images.

Authors’ original file for figure 1

Authors’ original file for figure 2

Authors’ original file for figure 3

Authors’ original file for figure 4

Authors’ original file for figure 5
