# Correction to: Genetic diversity of arsenic accumulation in rice and QTL analysis of methylated arsenic in rice grains

**DOI:** 10.1186/s12284-018-0221-6

**Published:** 2018-04-24

**Authors:** Masato Kuramata, Tadashi Abe, Akira Kawasaki, Kaworu Ebana, Taeko Shibaya, Masahiro Yano, Satoru Ishikawa

**Affiliations:** 1 Soil Environment Division, National Institute for Agro-Environmental Sciences, 3-1-3 Kannondai, Tsukuba, Ibaraki, 305-8604 Japan; 2 Genetic Resources Center, National Institute of Agrobiological Sciences, 2-1-2 Kannondai, Tsukuba, Ibaraki, 305-8602 Japan; 3 Agrogenomics Research Center, National Institute of Agrobiological Sciences, 2-1-2 Kannondai, Tsukuba, Ibaraki, 305-8602 Japan

## Correction

The authors of article “Genetic diversity of arsenic accumulation in rice and QTL analysis of methylated arsenic in rice grains” (Kuramata et al. 2013) would like to note that the original version of the article online unfortunately contains the following errors:

1) In Figure 2, “WRC13 (Tima)” should instead read “WRC13 (Asu)”.

2) In the ‘Results’ section, under the heading ‘QTL analysis for DMA concentrations of rice grains’ (PDF page 4, line 12 of the left column), the text “grain DMA concentrations (0.041-0.351 mg kg^-1^)” should instead read “grain DMA concentrations (0.027–0.106 mg kg^-1^)”.

3) In the ‘Discussion’ section (PDF page 7, lines 29-30 of the left column), the parentheses are not needed for “Zhang et al. 2008”.

4) Also in the ‘Discussion’ section (PDF page 7, line 9 of the right column), the parentheses are not needed for “Carey et al. 2010”.
