# Supplementary material for: Genetic diversity of arsenic accumulation in rice and QTL analysis of methylated arsenic in rice grains
Source: Rice (N Y). 2013 Jan 11;6:3. doi: 10.1186/1939-8433-6-3 (PMC5394917; doi:10.1186/1939-8433-6-3)
Supplement: Supplementary file 1 — Additional file 1: Inorganic As (iAs), DMA, and total As (tAs) concentrations in the grains of 58 cultivars grown for 3 years. (DOC 102 KB) [file 12284_2012_38_MOESM1_ESM.doc]

Additional file 1. Inorganic As (iAs), DMA, and total As (tAs) concentrations in the grains of 58 cultivars grown for 3 years.

|  |  | 2007 | | | 2008 | | | 2009 | | |
| --- | --- | --- | --- | --- | --- | --- | --- | --- | --- | --- |
| accession No. | Cultivar | iAs | DMA | tAs | iAs | DMA | tAs | iAs | DMA | tAs |
|  |  | (mg kg^-1^) | | | (mg kg^-1^) | | | (mg kg^-1^) | | |
| WRC 01 | NIPPONBARE | 0.108 | 0.023 | 0.180 | 0.061 | 0.010 | 0.105 | 0.181 | 0.022 | 0.206 |
| WRC 02 | KASALATH | 0.135 | 0.013 | 0.160 | 0.102 | 0.012 | 0.123 | 0.202 | 0.020 | 0.243 |
| WRC 03 | BEI KHE | 0.079 | 0.019 | 0.115 | 0.029 | 0.011 | 0.068 | 0.132 | 0.023 | 0.169 |
| WRC 04 | JENA035 | 0.105 | 0.012 | 0.123 | 0.050 | 0.007 | 0.063 | 0.129 | 0.021 | 0.157 |
| WRC 05 | NABA | 0.100 | 0.027 | 0.142 | 0.062 | 0.008 | 0.075 | 0.121 | 0.016 | 0.146 |
| WRC 06 | PULUIK　ARANG | 0.122 | 0.029 | 0.181 | 0.066 | 0.012 | 0.079 | 0.224 | 0.027 | 0.296 |
| WRC 07 | DAVAO1 | 0.099 | 0.027 | 0.190 | 0.033 | 0.009 | 0.092 | 0.174 | 0.018 | 0.201 |
| WRC 09 | RINSISAN　HONG MI | 0.132 | 0.012 | 0.157 | 0.156 | 0.008 | 0.181 | 0.181 | 0.010 | 0.207 |
| WRC 10 | QIU ZAO ZHANG | 0.086 | 0.020 | 0.157 | 0.047 | 0.011 | 0.118 | 0.134 | 0.015 | 0.153 |
| WRC 11 | JINGUOYIN | 0.109 | 0.020 | 0.147 | 0.095 | 0.014 | 0.108 | 0.147 | 0.015 | 0.178 |
| WRC 12 | DAHONGGU | 0.142 | 0.033 | 0.173 | 0.074 | 0.015 | 0.095 | 0.146 | 0.024 | 0.186 |
| WRC 13 | ASU | 0.119 | 0.021 | 0.250 | 0.043 | 0.017 | 0.149 | 0.248 | 0.031 | 0.293 |
| WRC 14 | IR 58 | 0.119 | 0.037 | 0.186 | 0.045 | 0.014 | 0.149 | 0.213 | 0.016 | 0.226 |
| WRC 15 | CO 13 | 0.076 | 0.030 | 0.150 | 0.029 | 0.012 | 0.096 | 0.134 | 0.019 | 0.162 |
| WRC 16 | VARYFUTSI | 0.137 | 0.026 | 0.180 | 0.075 | 0.011 | 0.105 | 0.160 | 0.019 | 0.191 |
| WRC 17 | JI　MU PO | 0.092 | 0.014 | 0.168 | 0.041 | 0.014 | 0.110 | 0.173 | 0.021 | 0.204 |
| WRC 18 | QINGYU (SEIYU) | 0.101 | 0.014 | 0.175 | 0.037 | 0.010 | 0.110 | 0.207 | 0.020 | 0.234 |
| WRC 19 | DENG PAO ZHAI | 0.143 | 0.045 | 0.261 | 0.080 | 0.015 | 0.143 | 0.187 | 0.030 | 0.247 |
| WRC 20 | TADUKAN | 0.119 | 0.027 | 0.165 | 0.049 | 0.014 | 0.126 | 0.137 | 0.027 | 0.230 |
| WRC 21 | SHWE NANG GYI | 0.073 | 0.030 | 0.132 | 0.032 | 0.011 | 0.100 | 0.102 | 0.012 | 0.154 |
| WRC 22 | CALOTOC | 0.164 | 0.025 | 0.198 | 0.104 | 0.013 | 0.144 | 0.146 | 0.020 | 0.222 |
| WRC 23 | LEBED | 0.183 | 0.037 | 0.198 | 0.103 | 0.020 | 0.131 | 0.123 | 0.021 | 0.177 |
| WRC 24 | PINULUPOT1 | 0.115 | 0.026 | 0.164 | 0.058 | 0.014 | 0.127 | 0.143 | 0.020 | 0.174 |
| WRC 25 | MUHA | 0.072 | 0.012 | 0.116 | 0.027 | 0.017 | 0.090 | 0.111 | 0.017 | 0.137 |
| WRC 26 | JHONA2 | 0.078 | 0.025 | 0.175 | 0.016 | 0.022 | 0.083 | 0.095 | 0.023 | 0.132 |
| WRC 27 | NEPAL8 | 0.067 | 0.012 | 0.079 | 0.039 | 0.014 | 0.100 | 0.081 | 0.012 | 0.121 |
| WRC 28 | JARJAN | 0.207 | 0.025 | 0.252 | 0.105 | 0.012 | 0.130 | 0.169 | 0.018 | 0.233 |
| WRC 29 | KALO DHAN | 0.104 | 0.014 | 0.098 | 0.064 | 0.013 | 0.083 | 0.133 | 0.030 | 0.202 |
| WRC 30 | ANJANA DHAN | 0.119 | 0.020 | 0.221 | 0.043 | 0.013 | 0.128 | 0.131 | 0.018 | 0.180 |
| WRC 31 | SHONI | 0.106 | 0.010 | 0.106 | 0.081 | 0.006 | 0.105 | 0.104 | 0.021 | 0.139 |
| WRC 32 | TUPA121-3 | 0.087 | 0.012 | 0.138 | 0.043 | 0.004 | 0.060 | 0.121 | 0.018 | 0.183 |
| WRC 33 | SURJAMUKHI | 0.085 | 0.015 | 0.114 | 0.034 | 0.006 | 0.036 | 0.099 | 0.012 | 0.129 |
| WRC 34 | ARC 7291 | 0.080 | 0.013 | 0.123 | 0.044 | 0.007 | 0.058 | 0.134 | 0.017 | 0.185 |
| WRC 35 | ARC 5955 | 0.186 | 0.021 | 0.239 | 0.100 | 0.013 | 0.080 | 0.202 | 0.026 | 0.296 |
| WRC 36 | RATUL | 0.199 | 0.013 | 0.252 | 0.118 | 0.010 | 0.099 | 0.214 | 0.015 | 0.297 |
| WRC 37 | ARC 7047 | 0.153 | 0.018 | 0.174 | 0.122 | 0.012 | 0.107 | 0.172 | 0.015 | 0.254 |
| WRC 38 | ARC 11094 | 0.181 | 0.024 | 0.224 | 0.127 | 0.015 | 0.155 | 0.168 | 0.026 | 0.255 |
| WRC 39 | BADARI DHAN | 0.171 | 0.022 | 0.226 | 0.121 | 0.016 | 0.119 | 0.190 | 0.022 | 0.242 |
| WRC 40 | NEPAL 555 | 0.242 | 0.032 | 0.297 | 0.101 | 0.016 | 0.103 | 0.266 | 0.024 | 0.312 |
| WRC 41 | KALUHEENATI | 0.186 | 0.026 | 0.218 | 0.138 | 0.019 | 0.132 | 0.182 | 0.018 | 0.197 |
| WRC 42 | LOCAL BASMATI | 0.068 | 0.028 | 0.112 | 0.017 | 0.023 | 0.032 | 0.058 | 0.035 | 0.081 |
| WRC 43 | DIANYU 1 | 0.141 | 0.016 | 0.178 | 0.066 | 0.012 | 0.081 | 0.177 | 0.019 | 0.190 |
| WRC 44 | BASILANON | 0.104 | 0.036 | 0.152 | 0.070 | 0.024 | 0.094 | 0.130 | 0.031 | 0.180 |
| WRC 45 | MA SHO | 0.097 | 0.039 | 0.187 | 0.044 | 0.019 | 0.063 | 0.125 | 0.022 | 0.163 |
| WRC 46 | KHAO NOK | 0.082 | 0.037 | 0.156 | 0.047 | 0.024 | 0.062 | 0.124 | 0.027 | 0.160 |
| WRC 47 | JAGUARY | 0.177 | 0.037 | 0.301 | 0.040 | 0.029 | 0.091 | 0.165 | 0.031 | 0.223 |
| WRC 48 | KHAU MAC KHO | 0.146 | 0.045 | 0.225 | 0.039 | 0.033 | 0.083 | 0.171 | 0.034 | 0.233 |
| WRC 49 | PADI PERAK | 0.082 | 0.064 | 0.178 | 0.024 | 0.043 | 0.078 | 0.114 | 0.047 | 0.194 |
| WRC 50 | REXMONT | 0.088 | 0.015 | 0.135 | 0.024 | 0.012 | 0.068 | 0.111 | 0.015 | 0.137 |
| WRC 51 | URASAN 1 | 0.192 | 0.038 | 0.262 | 0.052 | 0.020 | 0.120 | 0.200 | 0.027 | 0.271 |
| WRC 52 | KHAU TAN CHIEM | 0.127 | 0.038 | 0.179 | 0.032 | 0.020 | 0.080 | 0.228 | 0.037 | 0.332 |
| WRC 53 | TIMA | 0.052 | 0.027 | 0.119 | 0.008 | 0.020 | 0.032 | 0.065 | 0.030 | 0.112 |
| WRC 55 | TUPA 729 | 0.115 | 0.030 | 0.154 | 0.039 | 0.025 | 0.088 | 0.131 | 0.024 | 0.216 |
| WRC 57 | MILYANG 23 | 0.143 | 0.022 | 0.203 | 0.040 | 0.014 | 0.090 | 0.146 | 0.014 | 0.188 |
| WRC 64 | PADI KUNING | 0.064 | 0.028 | 0.126 | 0.015 | 0.013 | 0.065 | 0.092 | 0.021 | 0.131 |
| WRC 98 | DEE　JIAO　HUA　LUO | 0.101 | 0.012 | 0.168 | 0.019 | 0.013 | 0.085 | 0.159 | 0.019 | 0.206 |
| WRC 99 | HONG　CHEUH　ZAI | 0.098 | 0.013 | 0.180 | 0.019 | 0.014 | 0.082 | 0.145 | 0.018 | 0.194 |
| WRC 100 | VANDARAN | 0.186 | 0.030 | 0.278 | 0.128 | 0.038 | 0.118 | 0.162 | 0.030 | 0.243 |
