# Supplementary figures and images for: Genetic diversity of arsenic accumulation in rice and QTL analysis of methylated arsenic in rice grains
Source: Rice (N Y). 2013 Jan 11;6:3. doi: 10.1186/1939-8433-6-3 (PMC5394917; doi:10.1186/1939-8433-6-3)

## Slide 1
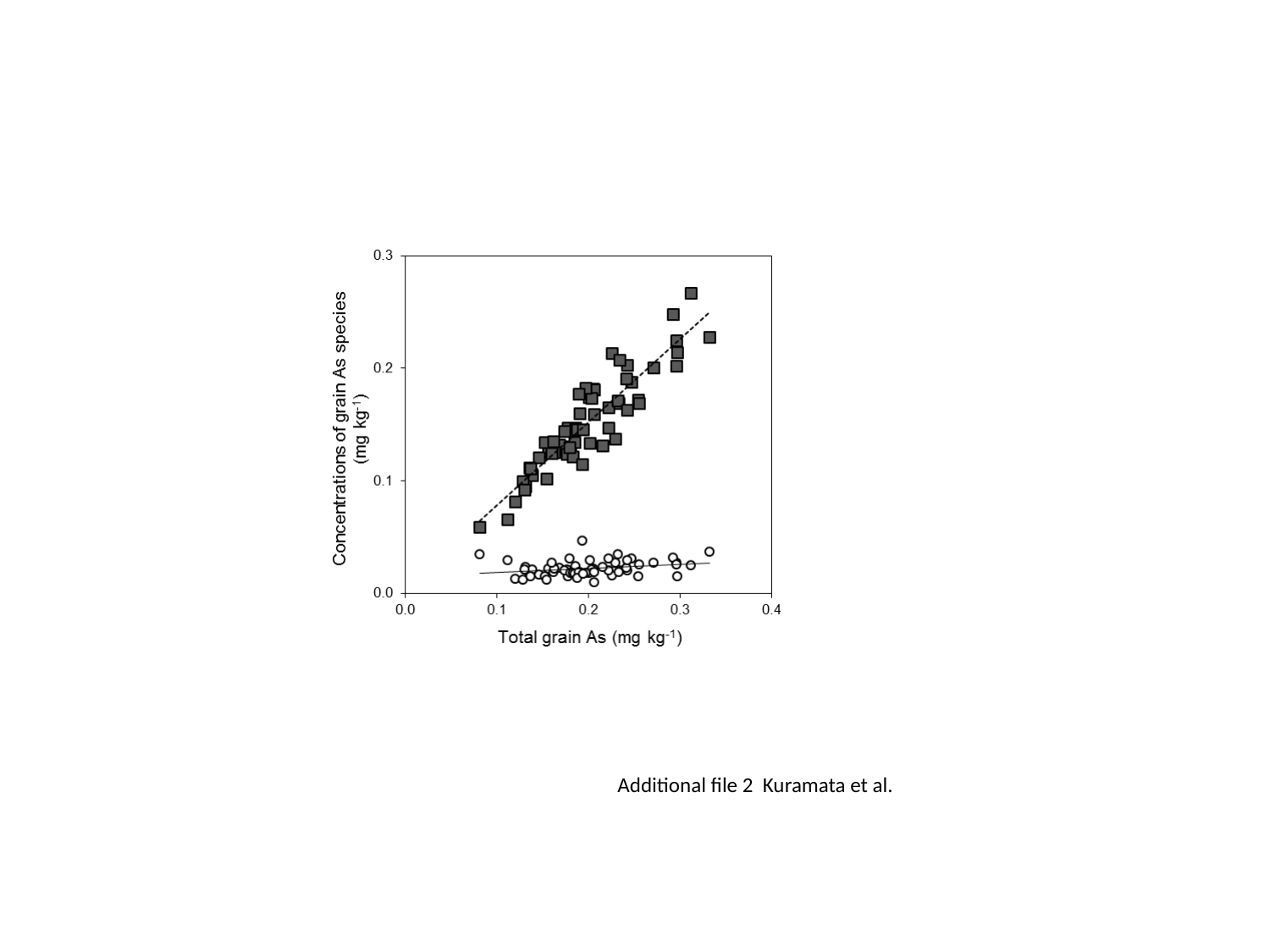

Additional file 2 Kuramata et al.

Supplement: Supplementary file 2 — Additional file 2: Correlation of total As and each the concentration of each As species in unpolished grains of WRC grown in 2009: filled square, inorganic As (r = 0.909, n = 58, p < 0.001); open circle, DMA (r = 0.270, n = 58, p < 0.05). (PPT 137 KB) [file 12284_2012_38_MOESM2_ESM.ppt]

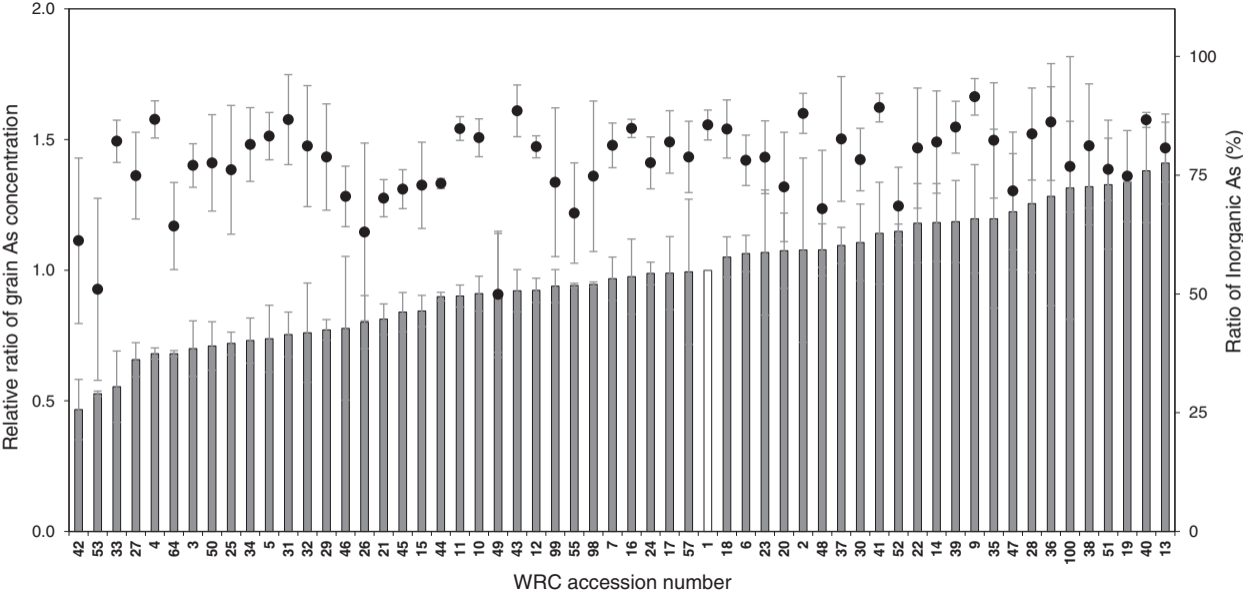

Supplement: Supplementary file 5 — Authors’ original file for figure 1 [file 12284_2012_38_MOESM5_ESM.pdf]

WRC1 (Nipponbare)

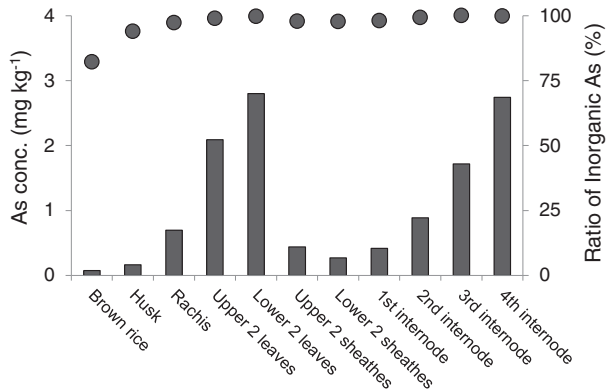

WRC13 (Tima)

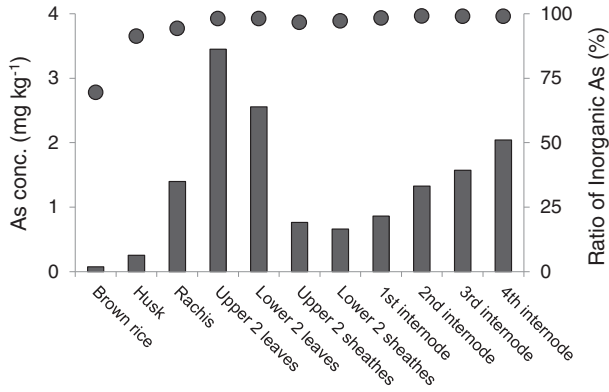

WRC42 (Local Basmati)

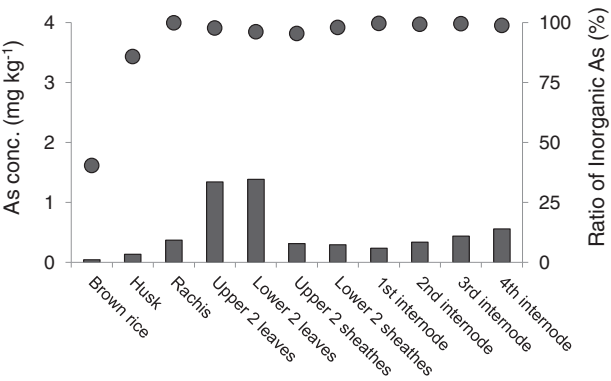

WRC49 (Padi Perak)

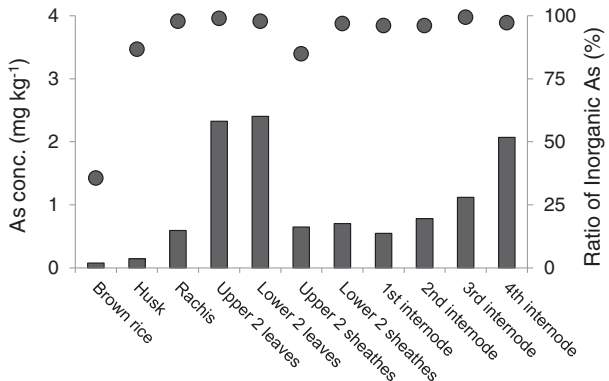

Supplement: Supplementary file 6 — Authors’ original file for figure 2 [file 12284_2012_38_MOESM6_ESM.pdf]

**A**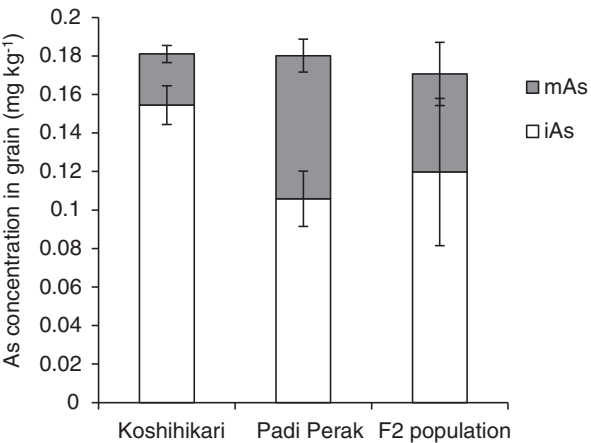**B**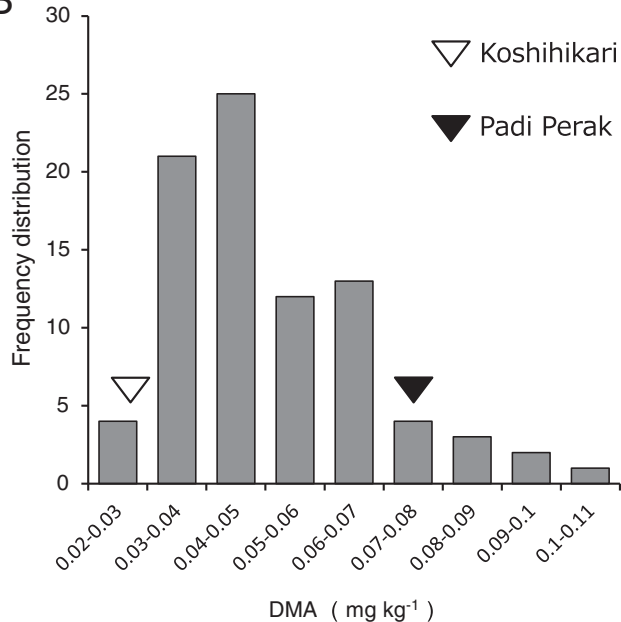

Supplement: Supplementary file 7 — Authors’ original file for figure 3 [file 12284_2012_38_MOESM7_ESM.pdf]

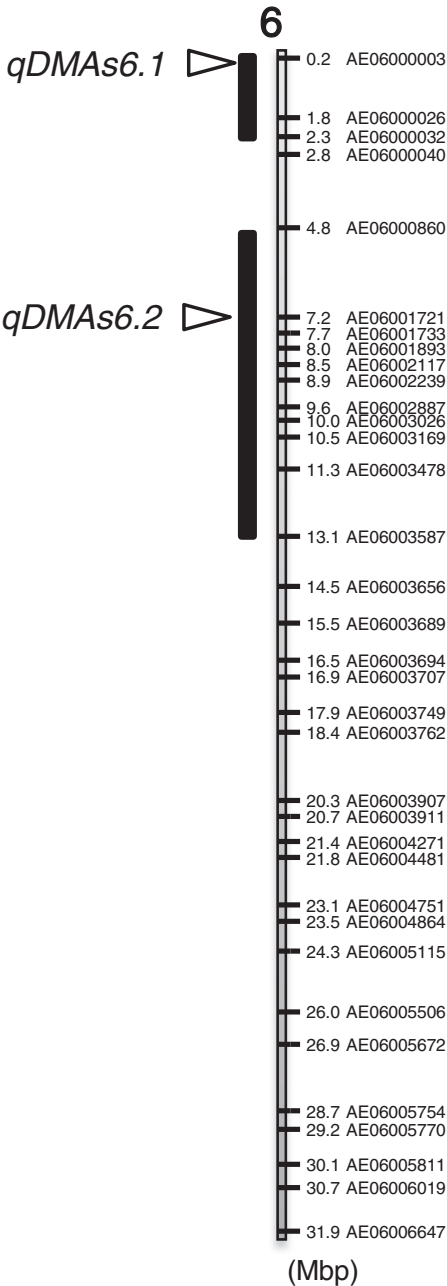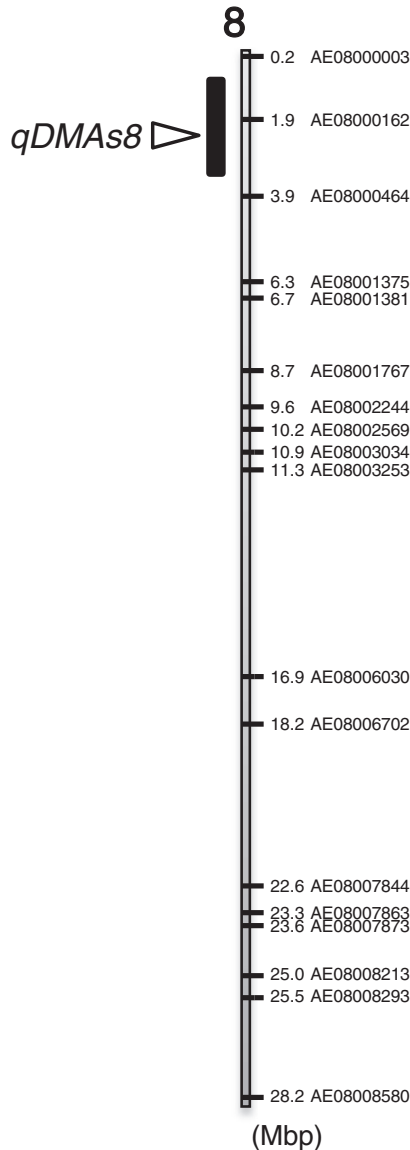

Supplement: Supplementary file 8 — Authors’ original file for figure 4 [file 12284_2012_38_MOESM8_ESM.pdf]

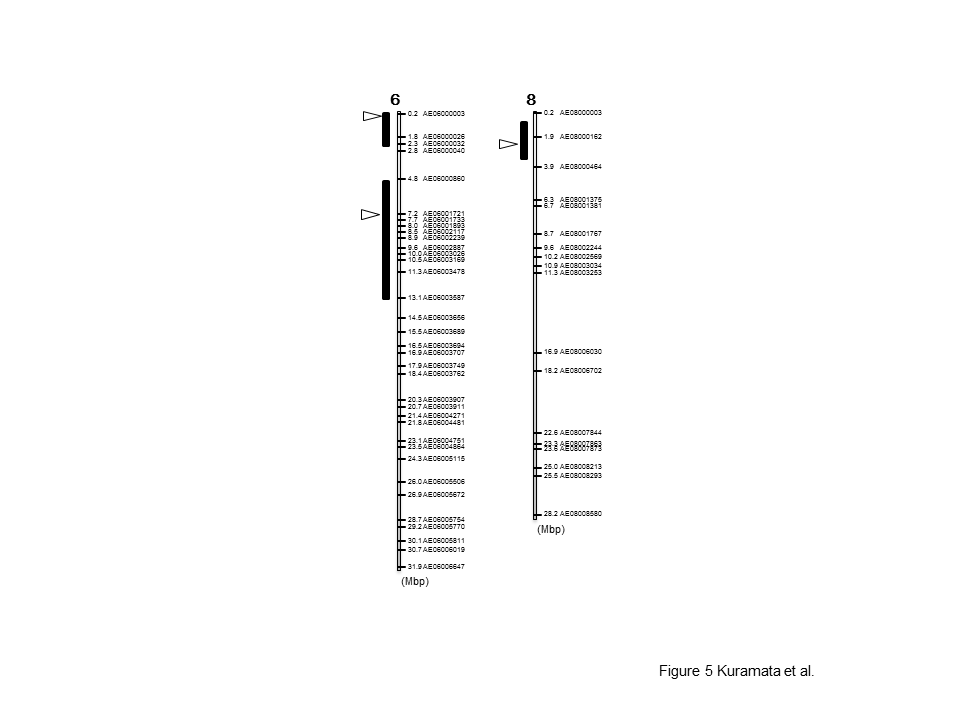

Supplement: Supplementary file 9 — Authors’ original file for figure 5 [file 12284_2012_38_MOESM9_ESM.tiff]
