# Supplementary material for: Genetic diversity of arsenic accumulation in rice and QTL analysis of methylated arsenic in rice grains
Source: Rice (N Y). 2013 Jan 11;6:3. doi: 10.1186/1939-8433-6-3 (PMC5394917; doi:10.1186/1939-8433-6-3)
Supplement: Supplementary file 4 — Additional file 4: Physical map constructed using 307 SNP markers. (PPT 3 MB) [file 12284_2012_38_MOESM4_ESM.ppt]

## Slide 1
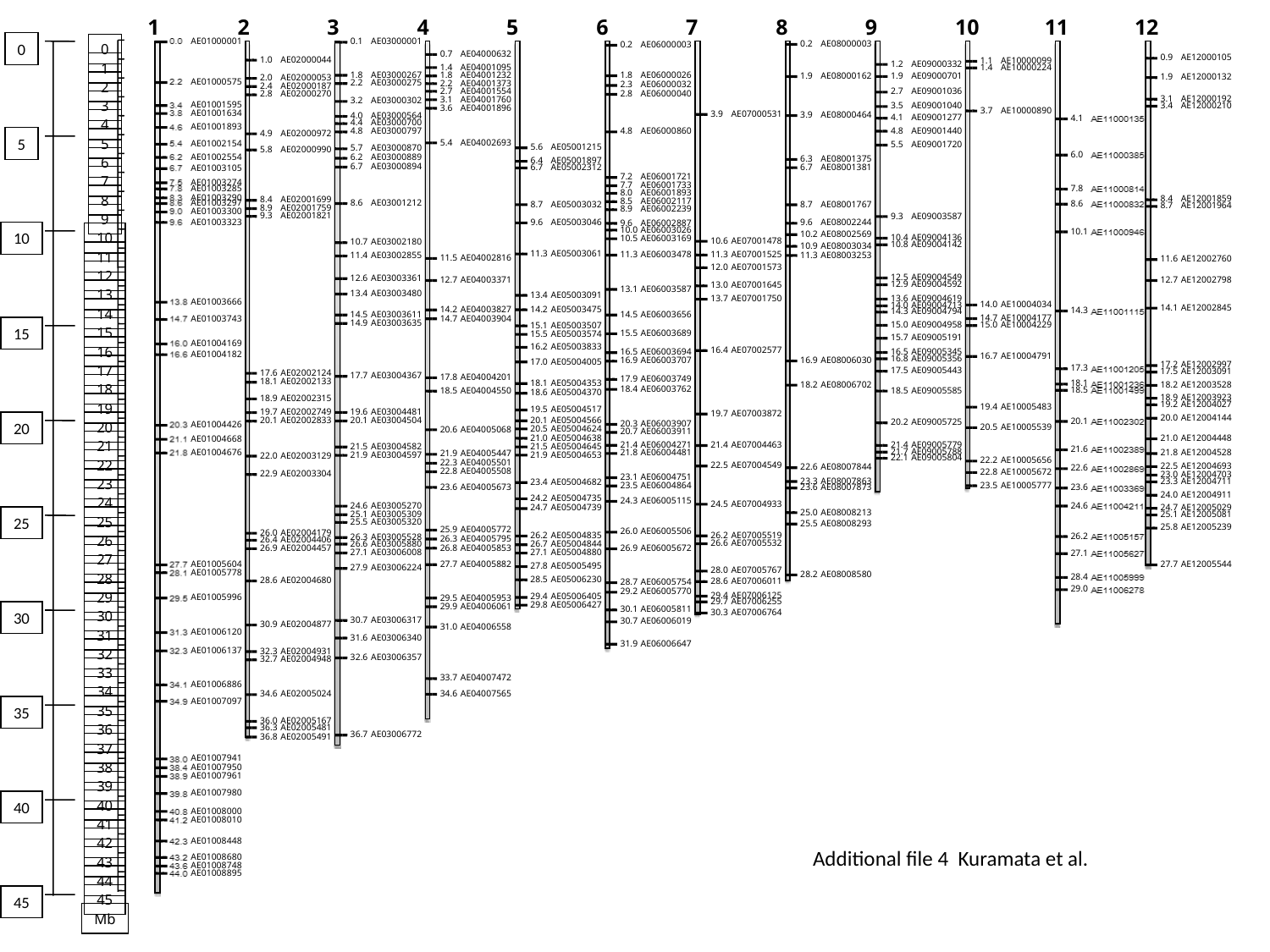

1
2
3
4
5
6
7
8
9
10
11
12
AE01000001
AE01000575
AE01001595
AE01001634
AE01001893
AE01002154
AE01002554
AE01003105
AE01003274
AE01003285
AE01003290
AE01003297
AE01003300
AE01003323
AE01003666
AE01003743
AE01004169
AE01004182
AE01004426
AE01004668
AE01004676
AE01005604
AE01005778
AE01005996
AE01006120
AE01006137
AE01006886
AE01007097
AE01007941
AE01007950
AE01007961
AE01007980
AE01008000
AE01008010
AE01008448
AE01008680
AE01008748
AE01008895
0.1
1.8
2.2
3.2
4.0
4.4
4.8
5.7
6.2
6.7
8.6
10.7
11.4
12.6
13.4
14.5
14.9
17.7
19.6
20.1
21.5
21.9
24.6
25.1
25.5
26.3
26.6
27.1
27.9
30.7
31.6
32.6
36.7
AE03000001
AE03000267
AE03000275
AE03000302
AE03000564
AE03000700
AE03000797
AE03000870
AE03000889
AE03000894
AE03001212
AE03002180
AE03002855
AE03003361
AE03003480
AE03003611
AE03003635
AE03004367
AE03004481
AE03004504
AE03004582
AE03004597
AE03005270
AE03005309
AE03005320
AE03005528
AE03005880
AE03006008
AE03006224
AE03006317
AE03006340
AE03006357
AE03006772
0
5
10
15
20
25
30
35
40
45
0.2
1.9
3.9
6.3
6.7
8.7
9.6
10.2
10.9
11.3
16.9
18.2
22.6
23.3
23.6
25.0
25.5
28.2
AE08000003
AE08000162
AE08000464
AE08001375
AE08001381
AE08001767
AE08002244
AE08002569
AE08003034
AE08003253
AE08006030
AE08006702
AE08007844
AE08007863
AE08007873
AE08008213
AE08008293
AE08008580
0.2
1.8
2.3
2.8
4.8
7.2
7.7
8.0
8.5
8.9
9.6
10.0
10.5
11.3
13.1
14.5
15.5
16.5
16.9
17.9
18.4
20.3
20.7
21.4
21.8
23.1
23.5
24.3
26.0
26.9
28.7
29.2
30.1
30.7
31.9
AE06000003
AE06000026
AE06000032
AE06000040
AE06000860
AE06001721
AE06001733
AE06001893
AE06002117
AE06002239
AE06002887
AE06003026
AE06003169
AE06003478
AE06003587
AE06003656
AE06003689
AE06003694
AE06003707
AE06003749
AE06003762
AE06003907
AE06003911
AE06004271
AE06004481
AE06004751
AE06004864
AE06005115
AE06005506
AE06005672
AE06005754
AE06005770
AE06005811
AE06006019
AE06006647
0
1
2
3
4
5
6
7
8
9
10
11
12
13
14
15
16
17
18
19
20
21
22
23
24
25
26
27
28
29
30
31
32
33
34
35
36
37
38
39
40
41
42
43
44
45
Mb
0.7
1.4
1.8
2.2
2.7
3.1
3.6
5.4
11.5
12.7
14.2
14.7
17.8
18.5
20.6
21.9
22.3
22.8
23.6
25.9
26.3
26.8
27.7
29.5
29.9
31.0
33.7
34.6
AE04000632
AE04001095
AE04001232
AE04001373
AE04001554
AE04001760
AE04001896
AE04002693
AE04002816
AE04003371
AE04003827
AE04003904
AE04004201
AE04004550
AE04005068
AE04005447
AE04005501
AE04005508
AE04005673
AE04005772
AE04005795
AE04005853
AE04005882
AE04005953
AE04006061
AE04006558
AE04007472
AE04007565
0.9
1.9
3.1
3.4
8.4
8.7
11.6
12.7
14.1
17.2
17.5
18.2
18.9
19.2
20.0
21.0
21.8
22.5
23.0
23.3
24.0
24.7
25.1
25.8
27.7
AE12000105
AE12000132
AE12000192
AE12000210
AE12001859
AE12001964
AE12002760
AE12002798
AE12002845
AE12002997
AE12003091
AE12003528
AE12003923
AE12004027
AE12004144
AE12004448
AE12004528
AE12004693
AE12004703
AE12004711
AE12004911
AE12005029
AE12005081
AE12005239
AE12005544
1.0
2.0
2.4
2.8
4.9
5.8
8.4
8.9
9.3
17.6
18.1
18.9
19.7
20.1
22.0
22.9
26.0
26.4
26.9
28.6
30.9
32.3
32.7
34.6
36.0
36.3
36.8
AE02000044
AE02000053
AE02000187
AE02000270
AE02000972
AE02000990
AE02001699
AE02001759
AE02001821
AE02002124
AE02002133
AE02002315
AE02002749
AE02002833
AE02003129
AE02003304
AE02004179
AE02004406
AE02004457
AE02004680
AE02004877
AE02004931
AE02004948
AE02005024
AE02005167
AE02005481
AE02005491
1.1
1.4
3.7
14.0
14.7
15.0
16.7
19.4
20.5
22.2
22.8
23.5
AE10000099
AE10000224
AE10000890
AE10004034
AE10004177
AE10004229
AE10004791
AE10005483
AE10005539
AE10005656
AE10005672
AE10005777
1.2
1.9
2.7
3.5
4.1
4.8
5.5
9.3
10.4
10.8
12.5
12.9
13.6
14.0
14.3
15.0
15.7
16.5
16.8
17.5
18.5
20.2
21.4
21.7
22.1
AE09000332
AE09000701
AE09001036
AE09001040
AE09001277
AE09001440
AE09001720
AE09003587
AE09004136
AE09004142
AE09004549
AE09004592
AE09004619
AE09004713
AE09004794
AE09004958
AE09005191
AE09005345
AE09005356
AE09005443
AE09005585
AE09005725
AE09005779
AE09005788
AE09005804
3.9
10.6
11.3
12.0
13.0
13.7
16.4
19.7
21.4
22.5
24.5
26.2
26.6
28.0
28.6
29.4
29.7
30.3
AE07000531
AE07001478
AE07001525
AE07001573
AE07001645
AE07001750
AE07002577
AE07003872
AE07004463
AE07004549
AE07004933
AE07005519
AE07005532
AE07005767
AE07006011
AE07006125
AE07006255
AE07006764
4.1
6.0
7.8
8.6
10.1
14.3
17.3
18.1
18.5
20.1
21.6
22.6
23.6
24.6
26.2
27.1
28.4
29.0
5.6
6.4
6.7
8.7
9.6
11.3
13.4
14.2
15.1
15.5
16.2
17.0
18.1
18.6
19.5
20.1
20.5
21.0
21.5
21.9
23.4
24.2
24.7
26.2
26.7
27.1
27.8
28.5
29.4
29.8
AE05001215
AE05001897
AE05002312
AE05003032
AE05003046
AE05003061
AE05003091
AE05003475
AE05003507
AE05003574
AE05003833
AE05004005
AE05004353
AE05004370
AE05004517
AE05004566
AE05004624
AE05004638
AE05004645
AE05004653
AE05004682
AE05004735
AE05004739
AE05004835
AE05004844
AE05004880
AE05005495
AE05006230
AE05006405
AE05006427
Additional file 4 Kuramata et al.
